# An Act for the Regulation of the Care and Treatment of Lunatics; with Explanatory Notes and Comments

**Published:** 1846-01

**Authors:** 


Art. IV.—*An Act for the Regulation of the Care and Treatment of Lunatics; with explanatory Notes and Comments.* Edited by Forbes Winslow, m.d.—*London,* 1845. 8vo, pp. 174.

This little work contains, (1) a history of the legislation on the subject of lunacy; (2) general observations on the recent act; (3) an elaborate and minute analysis of the act itself; and (4) an account of the present condition of lunacy in England and Wales, based upon the recent reports of the commissioners in lunacy. The appendix contains lists of all the public and private asylums for the insane in England and Wales, with the number of inmates in each, and various other matters of interest relating to the general subject. The work is unsusceptible of analysis, and requires no comment. It is indispensable to every one charged with the care of lunatics in any way, and will afford much valuable information to all who take an interest in the subject of lunacy or in lunatics.

